# Mussel-inspired Polydopamine-treated Copper Foil as a Current Collector for High-performance Silicon Anodes

**DOI:** 10.1038/srep30945

**Published:** 2016-08-17

**Authors:** Inseong Cho, Seokhyeon Gong, Danoh Song, Young-Gi Lee, Myung-Hyun Ryou, Yong Min Lee

**Affiliations:** 1 Department of Chemical and Biological Engineering, Hanbat National University, 125 Dongseodaero, Yuseong-gu, Daejeon 34158, Republic of Korea; 2 Nano Convergence Devices Research Department, Power Control Device Research Section, Electronics and Telecommunications Research Institute (ETRI), 218 Gajeongno, Yuseong-gu, Daejeon 34129, Republic of Korea

## Abstract

A new Cu current collector was prepared by introducing a mussel-inspired polydopamine coating onto a Cu foil surface to improve the electrochemical performance of a Si electrode. The polydopamine coating covalently bonded the polymeric binder (with hydroxyl functional groups) via a condensation reaction. The coating improved the adhesion strength between the Si composite electrode and the Cu current collector (245.5 N m^−1^, 297.5 N m^−1^, and 353.2 N m^−1^ for the Si electrodes based on bare Cu, polydopamine-treated Cu without thermal treatment, and polydopamine-treated Cu with thermal treatment, respectively). We demonstrate that the detachment between the Si composite electrode and the current collector plays an important role in determining the electrochemical performance of the Si electrode. The cycle life and rate capability of the Si electrode improved when the polydopamine surface-treated Cu current collector was used (963.9 mAh g^−1^, 1361.1 mAh g^−1^, and 1590.0 mAh g^−1^ for the Si electrodes based on bare Cu, polydopamine-treated Cu without thermal treatment, and polydopamine-treated Cu with thermal treatment, respectively, at C/2 after 500 cycles).

Lithium-ion batteries (LIBs) are used to power portable electric devices (e.g., cell phones, laptops, and cameras), but their application in electric vehicles (EVs) and energy storage systems (ESSs) requires significant increases in volumetric and gravimetric capacities, longer cycle lives, and lower costs^1^,^2^,^3^. Thus, electrodes that can deliver a larger amount of lithium ions than the current electrodes must be developed because the current electrodes have nearly reached their practical capacity limits^4^,^5^,^6^,^7^.

Si is a promising candidate for next-generation anode materials because of its extremely high theoretical specific capacity (approximately 4200 mAh g^−1^), relatively low operating potential, worldwide abundance, and environmentally friendly nature^4^. However, Si suffers from huge volume changes (approximately 400%) during the Li^+^ insertion and extraction processes, which causes the pulverization and cracking of the Si particles^4^,^8^. These morphological changes continually consume a large amount of Li ions and electrolytes to form a solid electrolyte interphase (SEI) layer on the newly exposed Si surface^9^,^10^. Furthermore, these changes break the electrical contact between the Si particles and the conductive additives^9^,^11^,^12^,^13^. The detached Si particles, which are called dead Si, are the major reason for the capacity loss during cycling^11^,^12^,^13^,^14^. Consequently, these factors result in a short shelf life for Si-based batteries, mainly because of the fast capacity decay, which impedes the successful implementation of Si-based anodes in LIBs.

The dimensional modification of Si active materials has been a major tactic to improve the cycle life of Si electrodes by reducing the dimensional stress that accompanies the Si volume changes. For example, Si does not cause cracking and pulverization below a critical size^15^. Consequently, many types of Si active materials have been developed, such as nanowires, nanotubes, nanoparticles, and nanosheets^16^,^17^. However, nano-sized materials are unfavorable to use in commercial electrode production processes because they cause low tap densities, handling difficulties, and safety problems^18^,^19^. Furthermore, the high surface area of nano-sized materials consumes a larger amount of Li ions to form the SEI layer in the formation step. Although nano/micro-hierarchical structured Si may be a good alternative^16^,^20^, this option does not appear appropriate for mass production because of the complexity of the structures and high production costs.

Developing a polymeric binder for Si electrodes is a practical and efficient approach to improve the cycle life of Si electrodes. Numerous alternative polymeric binders, such as poly (acrylic acid) (PAA)^11^,^21^,^22^, carboxymethyl cellulose^23^,^24^, alginate^25^, polyamide-imide, polyimide^26^, and polymers that form crosslinking networks^27^,^28^, have been proposed. These binders significantly improve the cycle life of Si electrodes by maintaining a tighter connection between the Si particles and the conductive additives than polyvinylidene difluoride, which is the conventional polymeric binder.

Detachment between the current collector and the composite electrodes, which consist of active materials, polymeric binders, and conductive additives, is considered another important electrode failure mechanism in conventional LIBs (their active materials (LiNi_0.5_Mn_1.5_O_4_^29^ and graphite^30^) are based on intercalation chemistry). The adhesion property in Si electrodes, where large volume changes occur, plays a more important role in the electrode performance considering the negligible volume changes of intercalation-based active materials. Despite numerous attempts to develop alternative Cu current collector systems, previous studies mainly focused on the structural modification of conventional current collectors (e.g., surface-roughened current collectors or porous current collectors^31^,^32^,^33^,^34^) to simply increase the contact area between the composite electrode and the current collectors.

We modified the surface properties of a conventional Cu current collector by using a mussel-inspired polydopamine coating technique to improve the inherent adhesion property between the composite electrode and the current collectors by altering the adhesion mechanism. Polydopamine can be used to modify the surface of a wide range of inorganic and organic materials and even metals^35^,^36^,^37^. This approach is efficient and economical because we use the conventional Cu current collectors, and it is environmentally friendly because water is the major solvent. We investigated the effect of polydopamine-modified Cu current collectors on the cycle performance of Si electrodes and quantitatively analyzed the adhesion property using a surface and interfacial cutting analysis system (SAICAS).

## Results

### Characterization of the polydopamine film on the Cu current collectors

**Figure 1:**
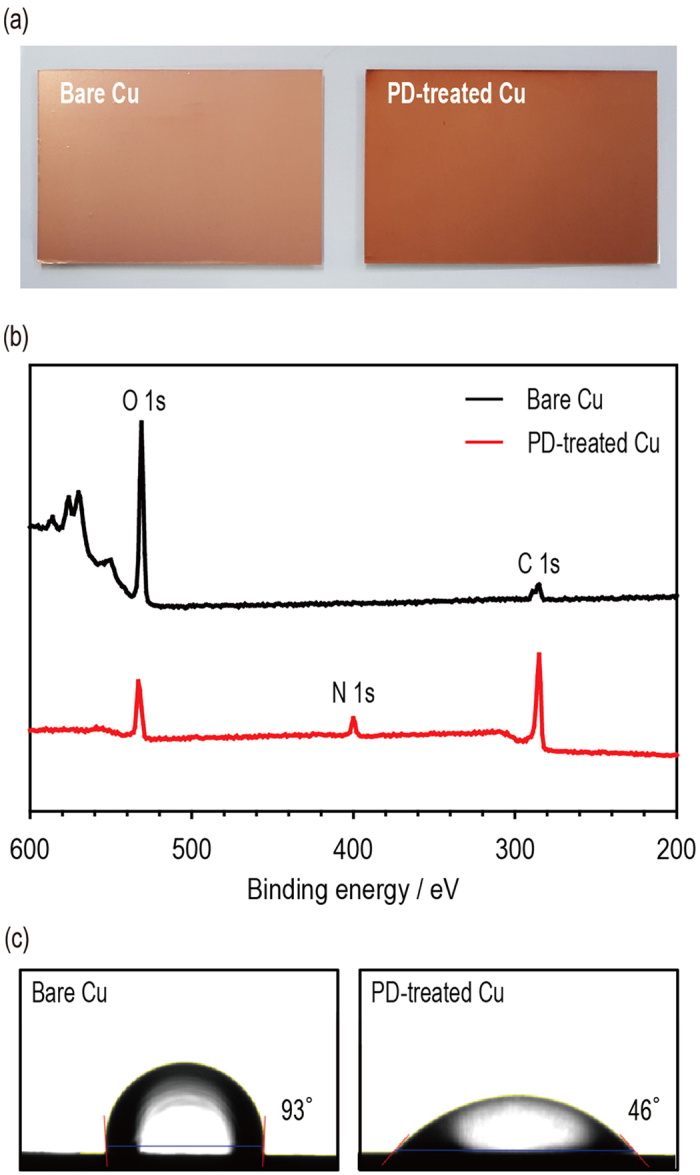
Preparation of the polydopamine-treated Cu current collector. (**a**) OM images of the bare Cu (left) and the polydopamine-treated Cu (right). (**b**) XPS spectra of the bare Cu (top) and polydopamine-treated Cu (bottom). (**c**) Contact angle images of the bare Cu (left) and the polydopamine-treated Cu (right).

After the polydopamine coating process, the polydopamine-treated Cu current collectors (PD-treated Cu) was more reddish-brown than the bare Cu current collectors (bare Cu) (Fig. 1a). The surfaces of the Cu current collectors were examined using X-ray photoelectron spectroscopy (XPS) to investigate the existence of polydopamine. As shown in Fig. 1b, the bare Cu provided a sharp O 1s peak, which was mainly attributed to the oxidized Cu and residual contaminants. After the polydopamine treatment, the XPS spectrum showed a new N 1s peak and a small O 1s peak. We infer that the polydopamine film was well formed on the Cu surfaces because polydopamine contains a large number of nitrogen-containing moieties^35^,^36^. See Supplementary Information for more detailed information (Figs S1 and S2).

In general, polydopamine can alter the surface from hydrophobic to hydrophilic. The surface properties of the bare Cu and PD-treated Cu were investigated by dropping a water droplet onto the surface of each current collector. As shown in Fig. 1c, the PD-treated Cu had a much smaller contact angle (46°) than did bare Cu (93°). Again, we attribute this result to the polar functional groups in polydopamine, which contains nitrogen and oxygen^35^,^36^.

### Preparation and characterization of the crosslinked PD-treated Cu

**Figure 2:**
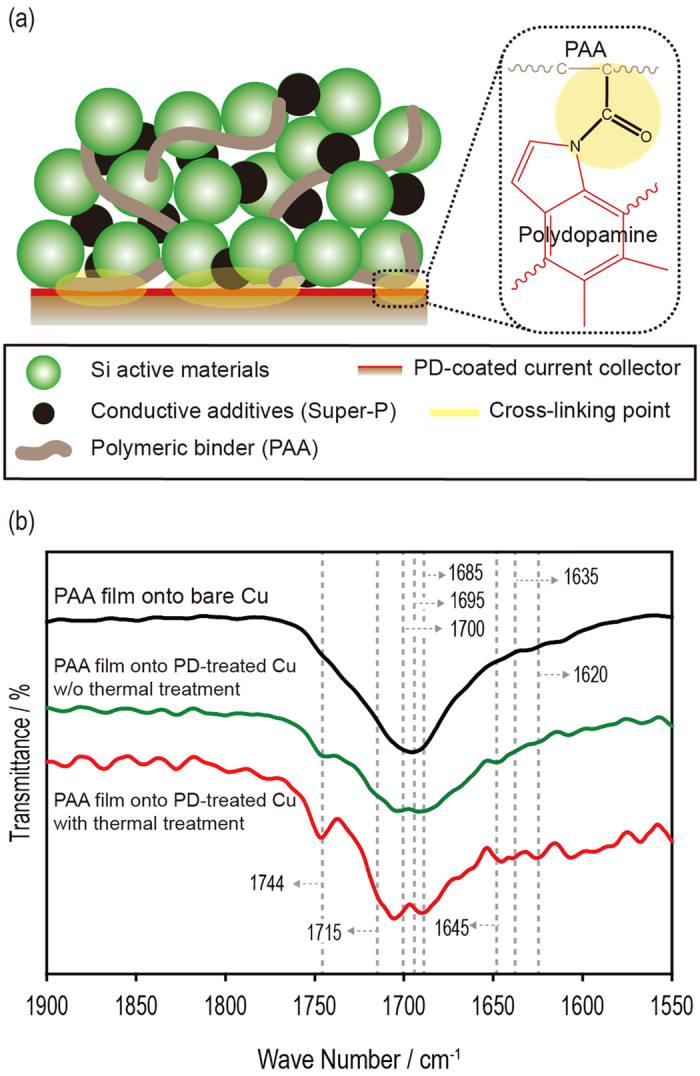
Characterization of the crosslinked PD-treated Cu. (**a**) Schematic for the covalent bonds between the PD-treated CU(X) -> Cu and the PAA binder in the Si composite electrode. (**b**) FTIR spectra of a PAA film coated onto bare Cu, a PAA film coated onto PD-treated Cu without thermal treatment, and a PPA film coated onto PD-treated Cu with thermal treatment (stored in a vacuum oven at 80 °C overnight).

The amino groups (N–H) can form covalent amide bonds with hydroxyl groups (O–H) via a condensation reaction^38^,^39^. Based on this reaction, we can chemically connect the polydopamine coating layer with a large number of amino groups (N–H) to the PAA polymeric binders, as illustrated in Fig. 2a. The covalent bonds help increase the adhesion strength between the polydopamine coating layer and the Si composite electrode.

Using FTIR, we investigated the PAA films that were coated onto bare Cu, PD-treated Cu without thermal treatment, and PD-treated Cu with thermal treatment to verify the presence of covalent bonding between the polydopamine coating layer and the PAA polymeric binder. For the thermal treatment, the sample was stored in a vacuum oven at 80 °C overnight. As shown in Fig. 2b, the PAA film on the Cu current collector showed a strong absorption peak at approximately 1695 cm^–1^, which is related to the carboxylate anion^40^,^41^ of the PAA polymeric binder. The PAA film on the PD-treated Cu had an absorption peak at 1645 cm^–1^, which is related to the N–H deformation vibration of polydopamine^42^. After the thermal treatment, the spectrum for the PAA film on the PD-treated Cu contained new peaks near 1744, 1715, 1700, 1685, 1635, and 1620 cm^−1^, which are related to the changes of the C = O groups of the amide^38^,^43^,^44^. These results are attributed to covalent bonding between the polydopamine coating layer and the PAA polymeric binder after the thermal treatment.

### Effect of PD-treated Cu current collectors on the electrochemical performance of a Si electrode

**Figure 3:**
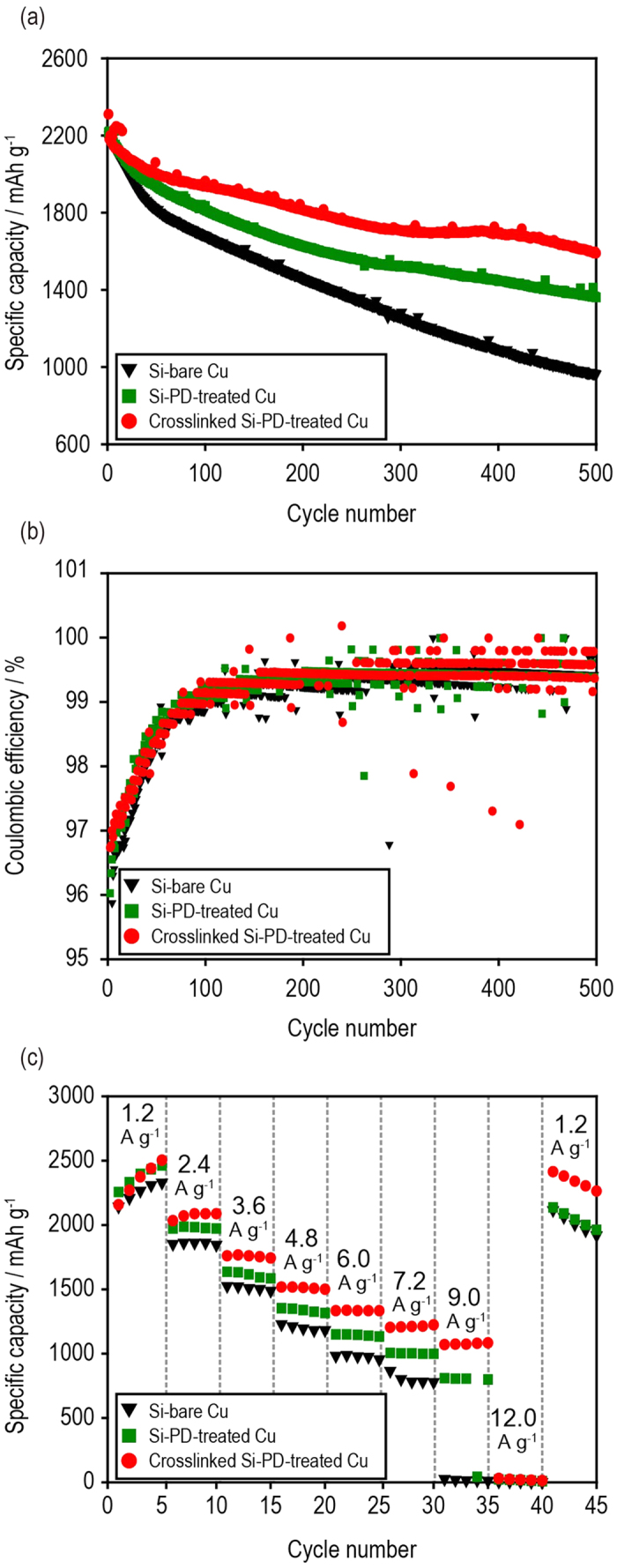
Electrochemical performance of the Si electrode. (**a**) Cycling performances (C/2, 1.2 A g^−1^), (**b**) coulombic efficiencies, and (**c**) the rate capabilities of the unit cells (Si/separator/Li metal, charged and discharged by changing the current density from C/2 (1.2 A g^−1^) to 5C (12 A g^−1^)).

We initially prepared Si electrodes using bare Cu (Si–bare Cu) and PD-treated Cu (Si–PD-treated Cu). Furthermore, some of the Si–PD-treated Cu received another thermal treatment (crosslinked Si–PD-treated Cu) by storing the sample in a vacuum oven at 80 °C overnight to form crosslinking networks between the polydopamine coating on the Cu current collectors and the PAA in the Si composite electrode. The effect of the PD-treated Cu on the electrochemical performance of the Si electrodes was investigated using 2032 coin-type half-cells (Si/separator/Li metal). As illustrated in Fig. 3a (see also Fig. S3 in Supplementary Information), the crosslinked Si–PD-treated Cu had the best discharge capacity retention ability (1590.0 mAh g^−1^; 69% of the initial discharge capacity) among the three Si electrodes after 500 cycles (operated at C/2 (1.2 A g^−1^)). The discharge capacity retention ability of the Si–PD-treated Cu and the Si–bare Cu electrodes were 1361.1 mAh g^−1^ (61% of the initial discharge capacity) and 963.9 mAh g^−1^ (44% of the initial discharge capacity), respectively. Similarly, each unit cell revealed the same coulombic efficiency (CE) trend during cycling performance. As illustrated in Fig. 3b, the crosslinked Si–PD-treated Cu had the highest CE among the three Si electrodes that reached 99.5%. This indicates a relatively stable SEI formation and minimal side reaction of the crosslinked Si–PD-treated Cu. See Fig. S4 in Supplementary Information for the cycle performances of Si electrodes having higher loading amount of 1 mg cm^−2^.

The effect of the PD-treated Cu on the rate capability of the Si electrode was also investigated using the identically prepared Si electrodes. The Si electrodes were charged and discharged by changing the current density from C/2 (1.2 A g^−1^) to 5C (12 A g^−1^). The experimental rate capability was similar to that obtained during the cycle performance, as shown in Fig. 3a. Again, the crosslinked Si–PD-treated Cu (1083.3 mAh g^−1^ at 9 A g^−1^) had a better rate capability than did both the Si–PD-treated Cu (800.0 mAh g^−1^ at 9 A g^−1^) and Si–bare Cu electrodes (6.7 mAh g^−1^ at 9 A g^−1^) (Fig. 3c).

### Morphological changes of the Si electrodes

**Figure 4:**
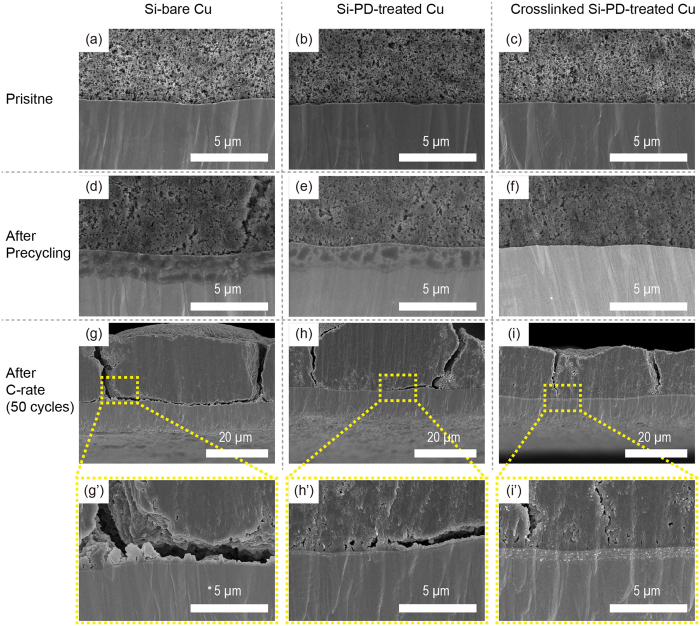
Morphological changes of the Si electrode. Cross-sectional images of Si-bare Cu, Si–PD-treated Cu, and crosslinked Si–PD-treated Cu (**a–c**) before cycling, (**d–f**) after precycling, and (**g–i’**) after the C-rate test.

We monitored the cross-sectional view of the Si electrodes using scanning electron microscopy (SEM) to investigate the morphological changes in the Si electrode during cycling. As shown in Fig. 4a–c, all three Si electrodes (Si–bare Cu, Si–PD-treated Cu, and crosslinked Si–PD-treated Cu) had similar morphological features before precycling. However, after precycling, the Si–bare Cu electrode had huge cracks across the Si composite, whereas the others did not show any huge cracks in the electrodes (Fig. 4d–f). The Si particles in all Si electrodes were enlarged after precycling. Several huge cracks were visible for all three Si electrodes after 50 rate-capability test cycles (Fig. 3b). A clear detachment between the Si composite and the Cu current collector appeared for the Si–bare Cu electrode (Fig. 4g,g’). In contrast, the Si–PD-treated Cu provided the Si composite with morphological features that were slightly detached from the PD-treated Cu (Fig. 4h,h’). Furthermore, the crosslinked Si–PD-treated Cu did not show any detachment between the Si composite and the PD-treated Cu (Fig. 4i,i’).

### Adhesion strength between the Si composite and the Cu current collectors

To investigate the relation between the physical properties of the Si composite and the electrochemical performance, we measured the adhesion strength of the Si electrodes using a SAICAS. Unlike the conventional peel test, a SAICAS can quantitatively measure the adhesion strength of the Si electrode at a specific depth from the surface by cutting the film using a V-shaped microblade^26^,^45^.

**Figure 5:**
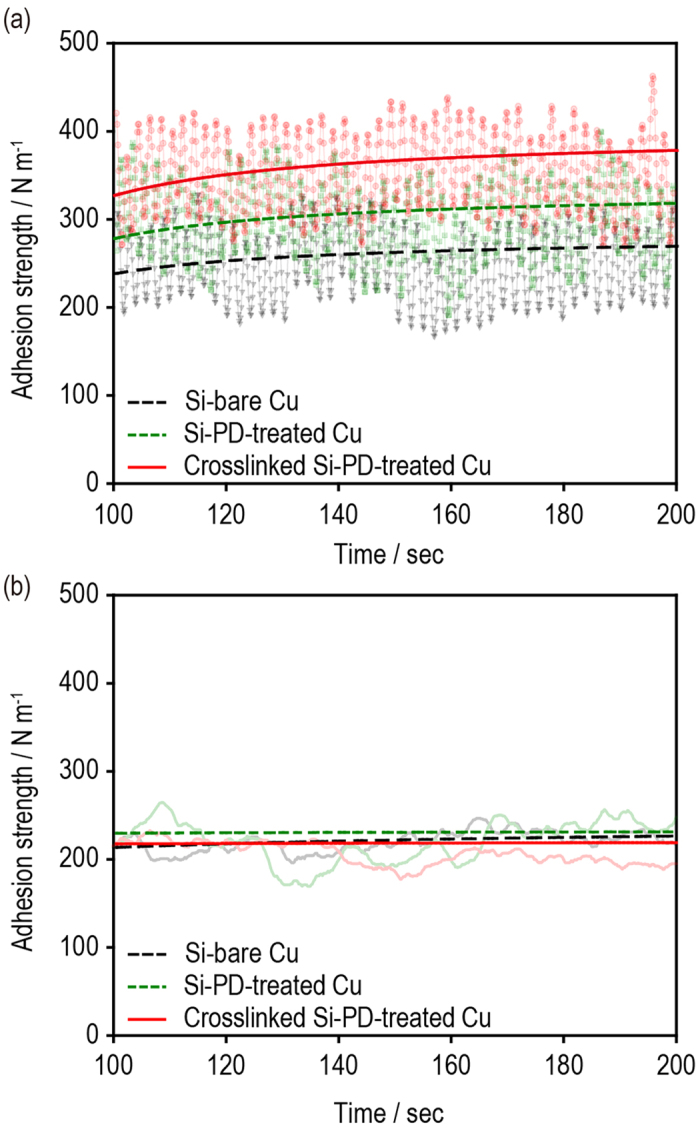
Adhesion strength between the Si composite and the Cu current collectors. (**a**) The adhesion strength between the Si composite and Cu current collector (*F*_*Si-Cu*_) and (**b**) the adhesion strength of the interlayer (*F*_*Si*_) at a specific depth of the Si composite (4 μm from the surface) measured using SAICAS.

Using SAICAS, we measured 1) the adhesion strength between the Si composite and the Cu current collector (*F*_*Si-Cu*_) and 2) the adhesion strength of the interlayer (*F*_*Si*_) at a specific depth of the Si composite (4 μm from the surface). As shown in Fig. 5a, the Si–PD-treated Cu had a larger *F*_*Si-Cu*_ than did the Si–bare Cu. Thus, the hydrogen bonding between polydopamine and PAA played a key role. In contrast, the crosslinked Si–PD-treated Cu had the highest *F*_*Si-Cu*_ (Si–bare Cu = 245.5 N m^−1^, Si–PD-treated Cu = 297.5 N m^−1^, and crosslinked Si–PD-treated Cu = 353.2 N m^−1^). This synergistic adhesion strength improvement may be attributed to the formation of a covalent bond between polydopamine and PAA during the thermal treatment, as previously discussed. In contrast, the Si electrodes had similar *F*_*Si*_ values (Si–bare Cu = 219.7 N m^−1^, Si–PD-treated Cu = 219.8 N m^−1^, and crosslinked Si–PD-treated Cu = 205.6 N m^−1^; Fig. 5b). These results are reasonable because the polydopamine coating layer on the Cu current collector rarely encounter O–H functional groups with the PAA in the mid layer of the Si composite electrode.

## Discussion

We demonstrated the feasibility of using a mussel-inspired polydopamine coating on a Cu current collector to improve the electrochemical performance of Si electrodes. Thus, we must consider the significant drawback of using polydopamine for this application because polydopamine is an insulating material, similar to most other polymeric materials. The insulating property of polydopamine may impede the current flow through the current collector and results in a poor electrochemical performance.

To investigate the effect of the polydopamine coating on the surface resistance of the Si electrodes (Si–bare Cu, Si–PD-treated Cu, and crosslinked Si–PD-treated Cu), we measured the resistances of 1) the outer layer of the Si composite electrode (*R*_*out*_) and 2) the interlayer between the Si composite electrode and the Cu current collector (*R*_*in*_) (Table 1). As expected, the Si–bare Cu electrode had the smallest *R*_*out*_ and *R*_*in*_ values (0.595 Ω cm and 2.37 mΩ cm^2^, respectively); the Si–PD-treated Cu (*R*_*out*_ = 3.48 Ω cm and *R*_*in*_ = 91.0 mΩ cm^2^) and crosslinked Si–PD-treated Cu (*R*_*out*_ = 3.65 Ω cm and *R*_*in*_ = 49.2 mΩ cm^2^) had higher *R*_*out*_ and *R*_*in*_ than the Si–bare Cu electrode. The easier electron transfer near the electrically conductive current collector makes *R*_*out*_ larger than *R*_*in*_for each Si electrode.

It is reasonable that the Si–PD-treated Cu (*R*_*out*_ = 3.48 Ω cm) and the crosslinked Si–PD-treated Cu (*R*_*out*_ = 3.65 Ω cm) have similar *R*_*out*_ values because they have a similar material constitution near the outside of the Si electrode even after the thermal treatment. In contrast, they had different *R*_*in*_ values (91.0 mΩ cm^2^ for the Si–PD-treated Cu and 49.2 mΩ cm^2^ for the crosslinked Si–PD-treated Cu). We believe that the reason for this is the change in material constitution in the chemical interaction between the Si composite electrode and the polydopamine layer during the thermal treatment.

In summary, although the polydopamine-related Si electrodes had higher surface resistances than the Si–bare Cu, we observed that the polydopamine insulating layer improved the electrochemical performance of the Si electrodes. We believe that the electrochemical performance of the Si electrode was affected by a combination of factors that accompany a larger volume change. Although the polydopamine coating layer enhanced the surface resistance of the Cu current collectors, it could transform the surface properties of the substrate from hydrophobic to hydrophilic. Moreover, the coating layer could form covalent bonds between the Si composite electrode and the Cu current collectors and improved their adhesion strength. The hydrophilic surface is advantageous when the material is wetted with polar liquid electrolytes, which are commonly used in commercialized LIBs. The improved adhesion strength between the Si composite electrode and the Cu current collector yielded better electrical connections during cycling, which improved the cycle performance and rate capability of the Si electrodes.

## Methods

### Preparation of the Polydopamine-treated Cu Current Collector

A Cu current collector was coated using the polydopamine (PD) coating process by Ryou *et al.*^37^ A mixture of a Tris buffer solution (pH 8.5, 10 mM) and methanol (CH_3_OH/buffer = 1/1 in wt%) was used to make a dopamine solution (2 mg mL^−1^).

### Electrode Preparation

Si electrodes were prepared from slurries that contained 60 wt% Si activated material (30 nm; Nanostructured & Amorphous Materials, Inc, Houston, USA), 20 wt% conductive carbon (Super-P, Timcal, Switzerland), and 20 wt% poly (acrylic acid) (MW = 450,000; Sigma-Aldrich, South Korea) binder in deionized water. The slurry was cast on two types of Cu current collectors: (i) a bare Cu current collector (8 μm; Iljin Materials, South Korea) and (ii) a Cu current collector that was treated with polydopamine on the surface of a bare Cu current collector (PD-treated Cu) using a doctor blade. The slurries that were cast onto the bare Cu and PD-treated Cu were dried in a convection oven at 80 °C in air for 2 h. In addition, the electrode with the PD-treated Cu was dried overnight in a vacuum oven at 80 °C to induce the crosslinking reaction between the PAA binder in the composite electrode material and the PD layer on Cu; the loading amount of the Si electrode was regulated to 0.53 mg cm^−2^ using a Mettler Toledo XP26 balance with an accuracy of 1 mg ± 0.001 (Fig. S5, Supplementary Information). The thicknesses of the three types of electrodes were controlled to around 8 μm confirmed by SAICAS (Fig. S5, Supplementary Information).

### Cell Assembly

The tested coin-2032 half cells were fabricated using Si anodes (diameter: 12 mm; dried in a vacuum oven at 60 °C for 12 h before use), Li metal as a counter electrode (450 μm; Honjo Metal Co., Japan), and a polyethylene separator (ND420, Asahi Kasei, Japan), which was impregnated with a liquid electrolyte (1.15 M LiPF_6_ in ethylene carbonate/ethyl methyl carbonate; EC/EMC = 3/7 vol%; Panax Etec, South Korea). The electrolyte contained 5 wt% fluoroethylene carbonate (Panax Etec, South Korea). The cell assembly processes were performed in an argon-filled glove box, where the dew point was maintained at less than −80 °C.

### Electrochemical Measurements

After the coin cells were stored for 12 h, a battery tester (PNE Solution, South Korea) was used to evaluate the formation step and cycle performance of the Si-bare Cu, Si–PD-treated Cu, and crosslinked Si–PD-treated Cu. The cells were cycled between 0.05 and 2.0 V at a constant current density of 0.2 A g^−1^ at room temperature. Then, the corresponding unit cells were cycled (between 0.05 and 2.0 V) at a current density of 1.2 A g^−1^ for 500 cycles to measure the cycle performance.

### Further Analysis

We confirmed that polydopamine was well coated on the surface of the Cu current collector using a contact angle analysis (Surface Electro Optics Co., Ltd., South Korea) and XPS (Sigma probe, Thermo VG Scientific, USA).

The attenuated total reflection FTIR (ATR-FTIR) spectra were recorded at a spectral resolution of 4 cm^−1^ in the frequency range of 600–4000 cm^−1^ (FTIR 4200, JASCO Co. Ltd., Japan) to observe the crosslinking reaction between the PD layer on the PD-treated Cu and the PAA binder in the composite electrode.

We cut and prepared three types of composite electrodes to confirm the contact between the electrode materials and the current collectors at three stages: (i) pristine state, (ii) after the formation step, and (iii) after 50 cycles. An ion milling system (E-3500, Hitachi, Japan) was used to cut each electrode at a constant power of 2.1 W (6 kV and 0.35 mA) in vacuum (<2.0 × 10^−4^ Pa). The cross-sectional morphologies of the electrodes were monitored using field-emission scanning electron microscopy (FE-SEM, S4800, Hitachi, Japan).

The internal adhesion strength in the composite electrode materials and interfacial adhesion strength between the electrodes and the Cu current collectors were measured using a surface and interfacial cutting analysis system (SAICAS, Daipla Wintes Co., Ltd., Japan). A boron nitride blade (blade width: 1 mm), which was fixed at a 45° shear angle, was used for the SAICAS measurements. During the test, the blade moved in the horizontal direction with a velocity of 2.0 μm s^−1^ while maintaining a vertical force of 0.2 N.

The volume resistances of the Si composite electrodes (outer layer resistances) and interfacial resistances between the composite electrodes and the current collectors (interlayer resistances) were measured using a multi-point-measuring-type electrode resistance meter (HIOKI KOREA Co., ltd., Japan).

## Additional Information

**How to cite this article**: Cho, I. *et al.* Mussel-inspired Polydopamine-treated Copper Foil as a Current Collector for High-performance Silicon Anodes. *Sci. Rep.* **6**, 30945; doi: 10.1038/srep30945 (2016).

## Supplementary Material

Supplementary Information

## Figures and Tables

**Table 1:** Resistance of the Si composite.

	Si–bare Cu	Si–PD-treated Cu	Crosslinking Si–PD-treated Cu
*R*_*out*_/Ω cm	0.595	3.48	3.65
*R*_*in*_/mΩ cm^2^	2.37	91.0	49.2
