# Supplementary material for: Mussel-inspired Polydopamine-treated Copper Foil as a Current Collector for High-performance Silicon Anodes
Source: Sci Rep. 2016 Aug 17;6:30945. doi: 10.1038/srep30945 (PMC4987668; doi:10.1038/srep30945)
Supplement: Supplementary Information [file srep30945-s1.doc]

Supplementary Information

**Mussel-inspired Polydopamine-treated Copper Foil as a Current Collector for High-performance Silicon Anodes**

Inseong Cho^†1^, Seokhyeon Gong ^†1^, Danoh Song^1^, Young-Gi Lee^2^, Myung-Hyun Ryou*, & Yong Min Lee*

^1^Department of Chemical and Biological Engineering, Hanbat National University, 125 Dongseodaero, Yuseong-gu, Daejeon 34158, Republic of Korea

^2^Nano Convergence Devices Research Department, Power Control Device Research Section, Electronics and Telecommunications Research Institute (ETRI), 218 Gajeongno, Yuseong-gu, Daejeon 34129, Republic of Korea

**Characterization of the polydopamine film on the Cu current collectors.** After that, we realized that the thickness of PD layer should be investigated. However, it was not easy to determine the exact thickness of PD layer coated on the Cu current collector since it’s too thin to be observed directly using such as cross-sectional scanning electron microscopy images. Alternatively, we measured the thickness of the coated polydopamine layer on the silicon wafer by surface profiler (Dektak 150, Veeco, Japan). As shown in Fig. S1, the difference between the average height of the values in the green band (Bare Si wafer side) minus the average height in the red band (PD-treated Si wafer side) was approximately 16 nm. Additionally, the surface SEM image of PD-treated Cu showed not remarkable differences before and after PD treating on the copper current collector because it’s nano-scaled thin layer (Fig. S2), which confirmed the thickness of the coated polydopamine layer on the silicon wafer by surface profiler (Dektak 150, Veeco, Japan).


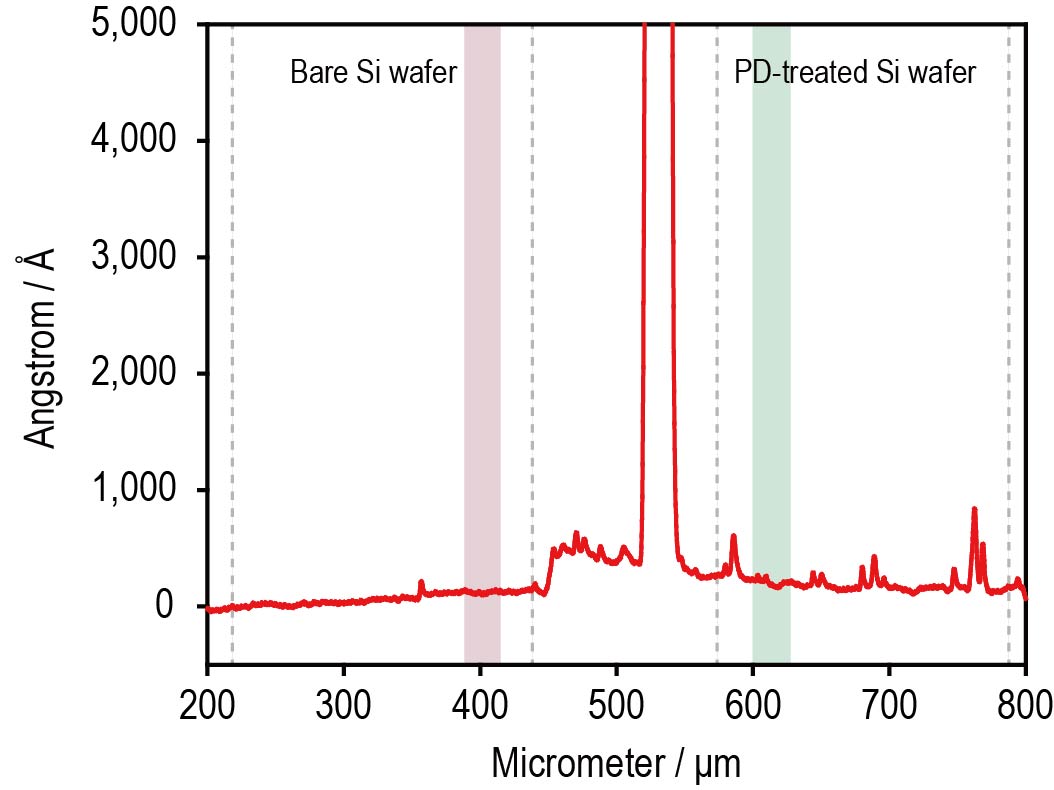


Figure S1. Surface profiles of the Si wafer and the PD-treated Si wafer.


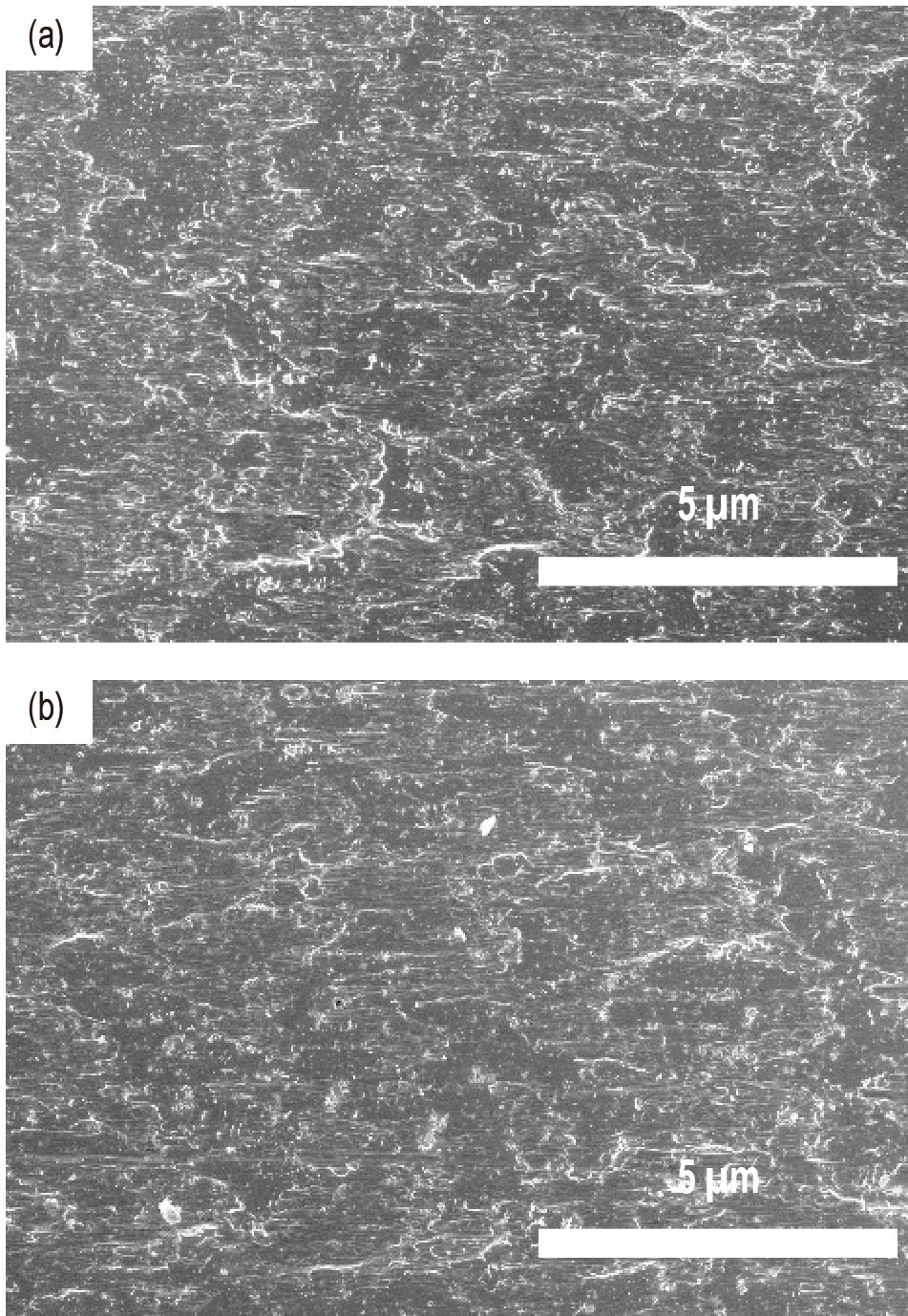


Figure. S2. Surface SEM images of (a) bare-Cu and (b) PD-treated Cu.


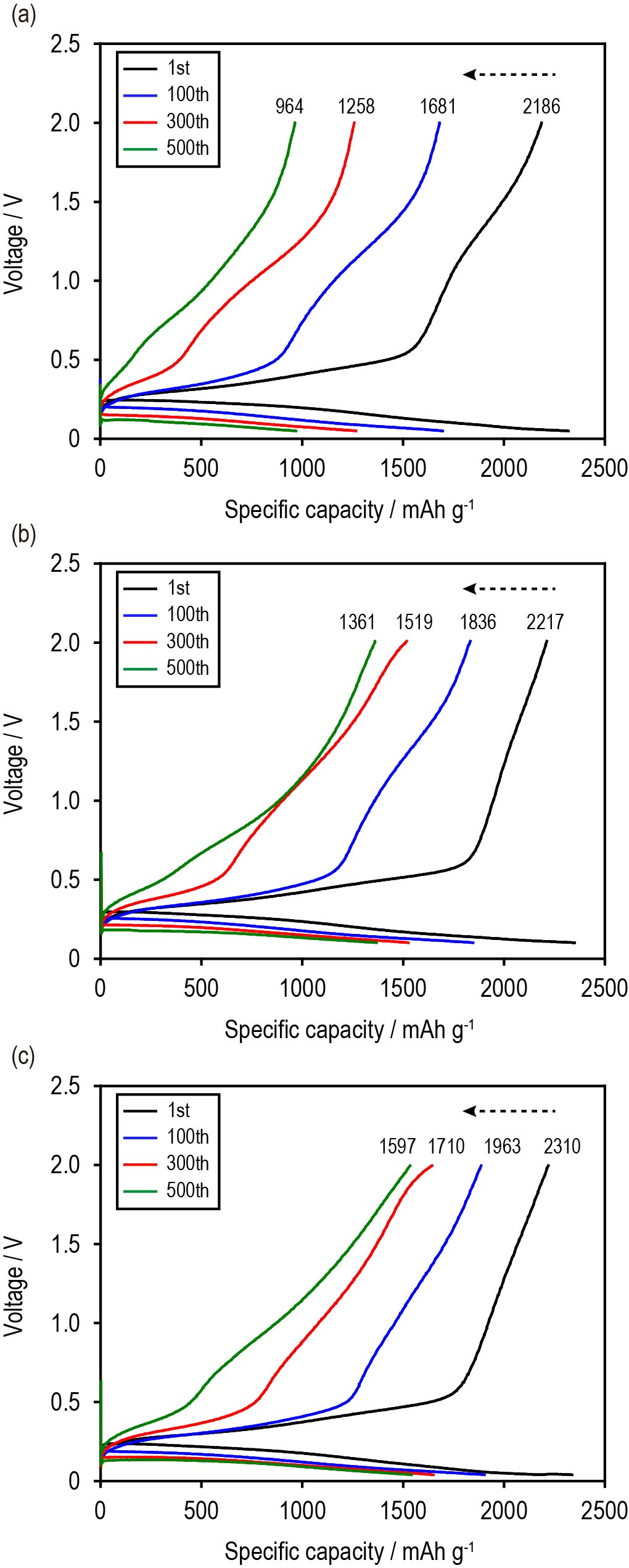


Figure S3. The plots of the galvanostatic cycling of (a) Si–bare Cu, (b) Si–PD-treated Cu, and (c) crosslinked Si–PD-treated Cu.


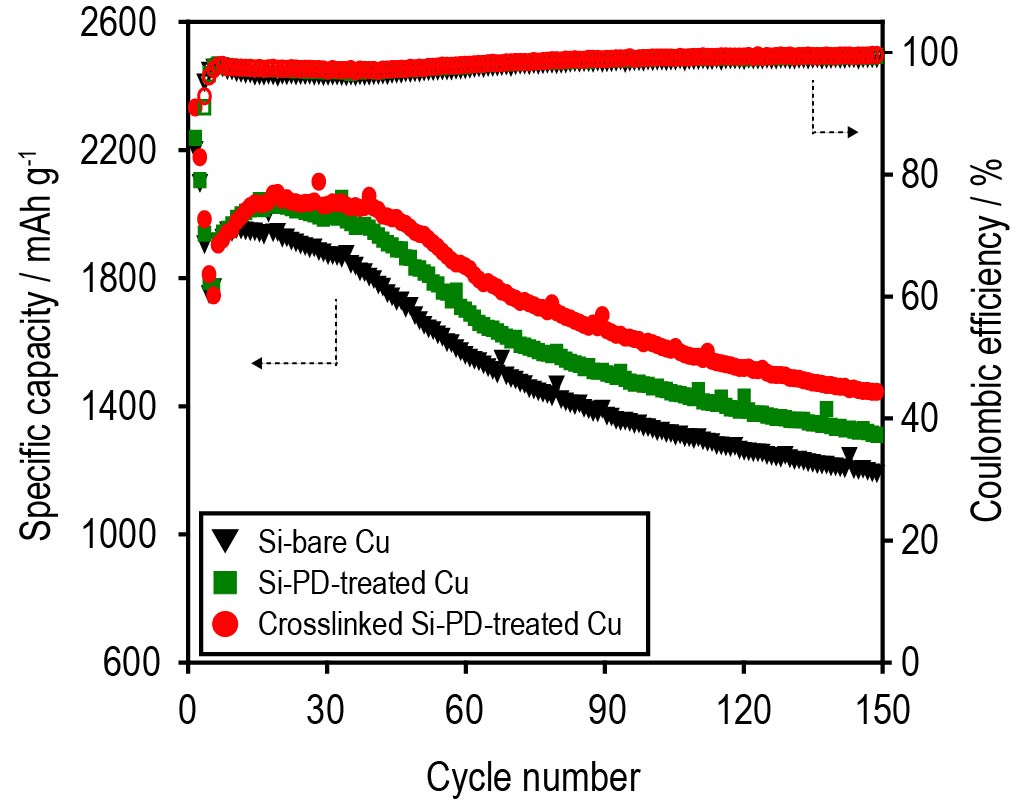


**Figure S4.** Cycling performances (C/2, 1.2 A g^-1^) of the unit cells (Si/separator/Li metal).


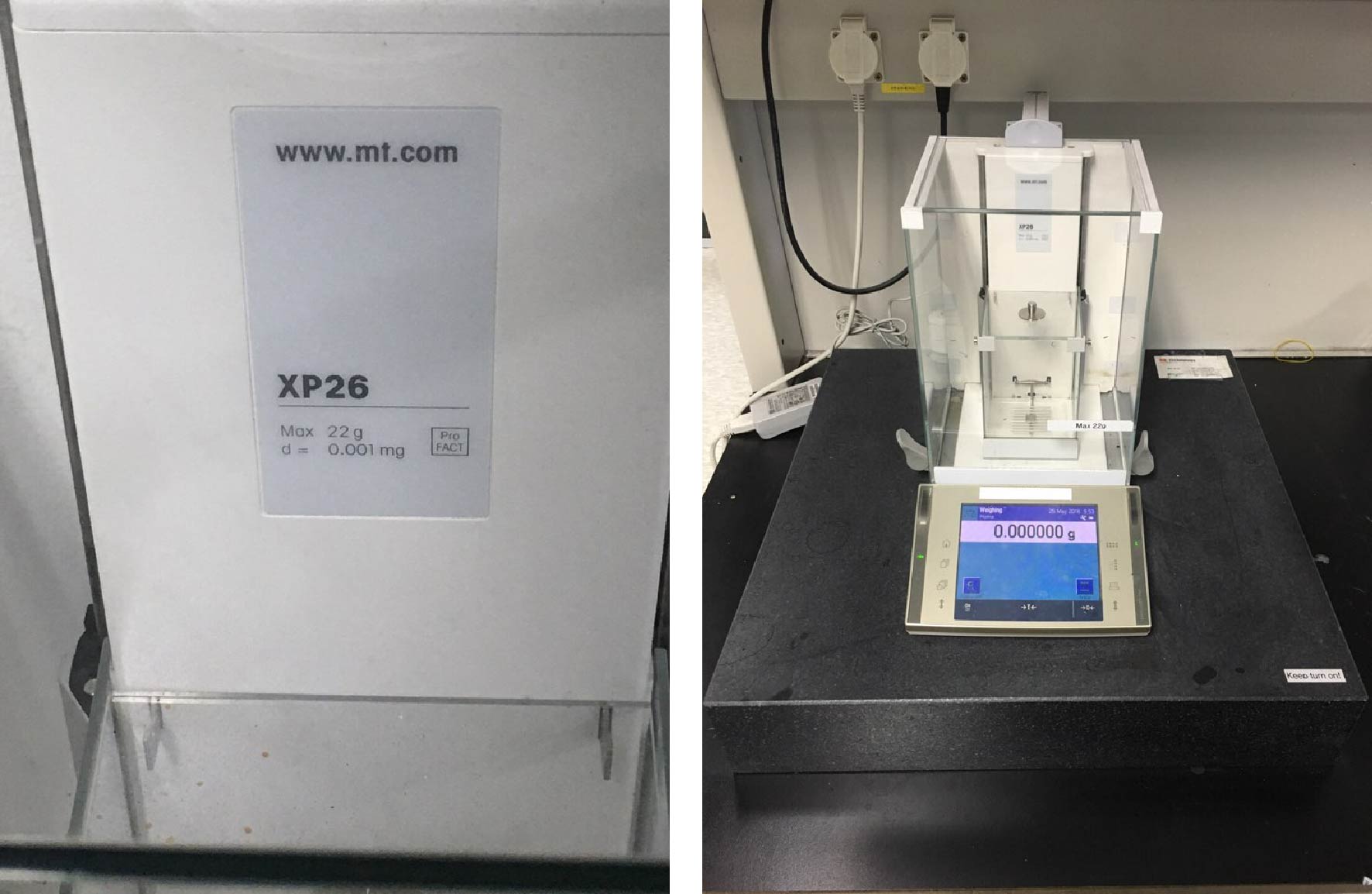


**Figure S5.** Laboratory balance used in the experiments of our study.


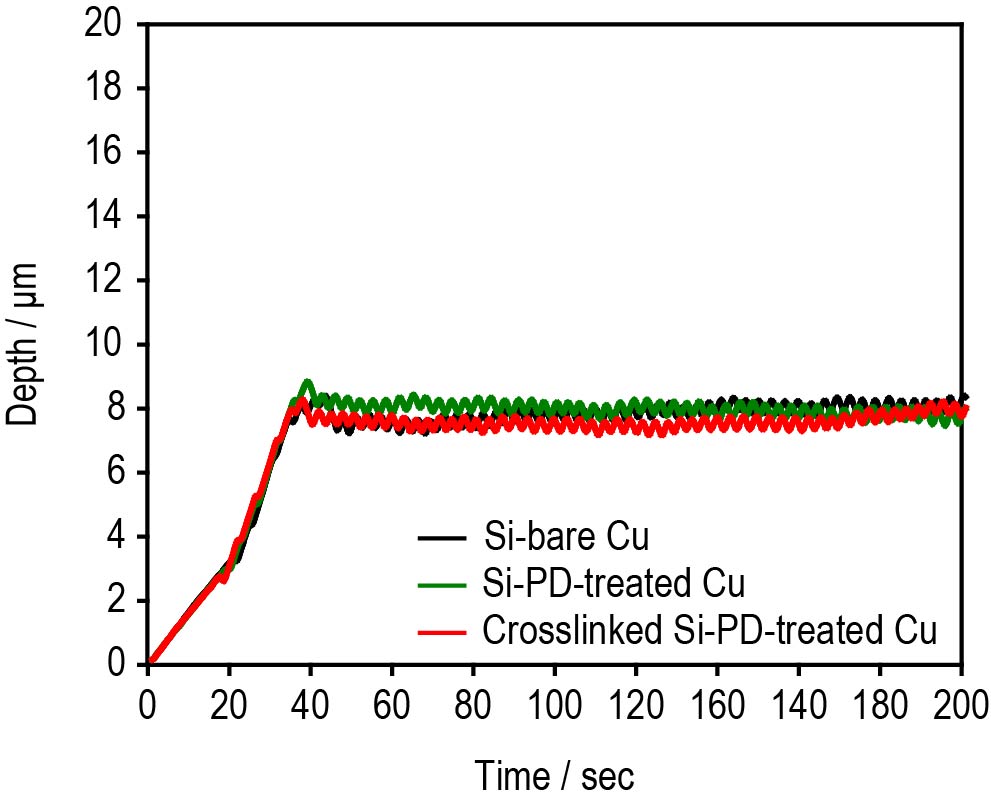


Figure S6. Electrode depth profiles of (a) Si–bare Cu, (b) Si–PD-treated Cu, and (c) crosslinked Si–PD-treated Cu.
